# Optimizing Non-invasive Oxygenation for COVID-19 Patients Presenting to the Emergency Department with Acute Respiratory Distress: A Case Report

**DOI:** 10.5811/cpcem.2020.6.48456

**Published:** 2020-06-22

**Authors:** David Zodda, Allyson Hanson, Alyssa Berns

**Affiliations:** * Hackensack University Medical Center School of Medicine at Seton Hall, Nutley, New Jersey; † Hackensack University Medical Center, Department of Emergency Medicine, Hackensack, New Jersey

**Keywords:** COVID-19, Hypoxia, Non-invasive, Oxygenation, Airway

## Abstract

**Introduction:**

The novel coronavirus (COVID-19) pandemic has led to an increase in the number of patients presenting to the emergency department (ED) with severe hypoxia and acute respiratory distress. With limited resources and ventilators available, emergency physicians working at a hospital within the epicenter of the United States outbreak developed a stepwise, non-invasive oxygenation strategy for treating COVID-19 patients presenting with severe hypoxia and acute respiratory distress.

**Case Report:**

A 72-year-old male suspected of having the COVID-19 virus presented to the ED with shortness of breath. He was found to be severely tachypneic, febrile, with rales in all lung fields. His initial oxygen saturation registered at SpO_2_ (blood oxygenation saturation) 55% on room air. Emergency physicians employed a novel non-invasive oxygenation strategy using a nasal cannula, non-rebreather, and self-proning. This approach led to a reversal of the patient’s respiratroy distress and hypoxia (SpO2 88–95%) for the following 24 hours. This non-invasive intervention allowed providers time to obtain and initiate high-flow nasal cannula and discuss end-of-life wishes with the patient and his family.

**Conclusion:**

Our case highlights a stepwise, organized approach to providing non-invasive oxygenation for COVID-19 patients presenting with severe hypoxia and acute respiratory distress. This approach primarily employs resources and equipment that are readily available to healthcare providers around the world. The intent of this strategy is to provide conventional alternatives to aid in the initial airway management of confirmed or suspected COVID-19 patients.

## INTRODUCTION

The novel coronavirus disease (COVID-19) pandemic, caused by the highly contagious severe acute respiratory syndrome-coronavirus-2 (SARS-CoV-2), is currently threatening the global human population.^1^ We describe the case of a 72-year-old male presenting to the emergency department (ED) with acute respiratory distress. The case described took place during the 2020 COVID-19 pandemic and occurred at an academic medical center with one of the highest rates of COVID-19 infections in the United States.

The COVID-19 pandemic has led to an increase in the number of patients presenting to the hospital with severe hypoxia and acute respiratory distress. We employed a novel non-invasive oxygensation strategy using a nasal cannula (NC), non-rebreather (NRB), and self-proning. This technique led to an improvement in the patient’s hypoxia and a 24-hour reversal in his acute respiratory failure, which allowed us time to obtain and initiate high-flow NC and discuss end-of-life wishes with the patient and his family.

Our case highlights a stepwise, organized approach to providing non-invasive oxygenation for hypoxic patients in acute respiratory distress. It was developed during the initial days of the COVID-19 pandemic at a frontline hospital at the epicenter of the US outbreak. This approach employs resources and equipment readily available to healthcare providers around the world. The intent of this strategy is to provide conventional alternatives to aid in the initial management of confirmed or suspected COVID-19 patients with acute hypoxic respiratory failure.

## CASE REPORT

A 72-year-old male presented to the ED for shortness of breath that had progressed over the prior seven days. He was found to be tachypneic, febrile, and with rales in all lung fields. His blood pressure and heart rate were within acceptable limits. He was awake, alert and cooperative. However, his oxygen saturation upon presentation to the ED was abnormal and initially registered at 55% on room air. The patient was placed on a cardiac monitor with pulse oximetry, and we obtained a chest radiograph revealing multilobar pneumonia (Image).

We employed a non-invasive oxygenation strategy using a NC at 6 liters per minute (LPM) and a NRB mask at 15 LPM. In addition, the patient was assisted to the prone position on the stretcher. Within five minutes, his mental status and work of breathing improved, his oxygen saturation improved to 95%, and he remained between 88–95% for the next 16 hours. Approximately 16 hours after arrival, the patient became hypoxic with oxygen saturations dipping below 88%. The NC was removed and a high-flow nasal cannula (HFNC) 60 LPM was added. Using this strategy of a HFNC, NRB, and self-proning, the patient remained alert and his oxygen saturation remained between 88–95% for a total of 24 hours.

**CPC-EM Capsule**

What do we already know about this clinical entity?

A common presentation of symptomatic patients with coronavirus disease 2019 (COVID-19) is severe hypoxia and respiratory distress often requiring emergent intubation in the emergency department (ED) setting.

What makes this presentation of disease reportable?

We present a COVID-19 patient presenting to the ED with profound hypoxia and respiratory distress and rather than intubate, we employed a novel stepwise approach to non-invasive oxygenation.

What is the major learning point?

A stepwise approach to non-invasive oxygenation of COVID -19 patients can delay emergent intubation, allow time for additional ventilatory treatments to become available, and provide time for emergency providers to clarify goals of care with patients and their family.

How might this improve emergency medicine practice?

Resuscitating the hypoxic COVID-19 patient is uniquely challenging and necessitates a stepwise approach for both provider safety and patient care.

Eventually, his mental status waned, his work of breathing became labored, and his oxygen saturation further deteriorated. Discussions with the patient and his family regarding advance directives revealed that his wishes were to be full code. He was admitted to the intensive care unit (ICU) and soon placed on a ventilator. Ultimately, our non-invasive strategy did not reverse this patient’s respiratory failure. However, it did allow us time (24 hours) to stabilize him, procure additional resuscitative resources, and discuss advance directives with him and his family.

## DISCUSSION

We present a stepwise approach to providing non-invasive oxygenation to confirmed or suspected COVID-19 patients presenting to the ED with hypoxia and acute respiratory distress. This strategy was developed during the initial days of the COVID-19 pandemic at a frontline hospital at the epicenter of the US outbreak. As in the case of our patient, this approach can be used to improve oxygen saturation, work of breathing, and may reduce the need for early mechanical ventilation.

Our pathway does not include non-invasive positive pressure ventilation (NIPPV). We did not have masks or helmets available that would have sufficiently protected staff from aerosolization of the COVID-19 virus during NIPPV. In addition, due to limited resources, we needed to repurpose all of our bi-level positive airway pressure machines into ventilators.

### Step 1: Patient and Provider Safety

Patients presenting to the ED during the COVID-19 pandemic with symptoms such as fever, dyspnea, and hypoxia should be suspected of having the COVID-19 virus.^2^ These patients should be provided with a surgical mask and placed in a single-occupancy, negative pressure room with a closed door.^3^

Contact and droplet precautions should be initiated for all patients suspected of having COVID-19, and providers should wear personal protective equipment (PPE) that includes a gown, gloves, eye protection, and a respirator (e.g., an N95 respirator).^4^ Healthcare providers should pay special attention to the appropriate sequence of putting on (donning) and taking off (doffing) of PPE to avoid contamination.

### Step 2: Initial Assessment

Patients presenting to the ED with confirmed or suspected COVID-19 should receive an initial assessment that includes evaluation of their airway, breathing, and circulation. The patient should be placed on a cardiac monitor to evaluate blood pressure, heart rate, and breaths per minute. Continuous pulse oximetry should be obtained. However, the presence of hypoxemia alone should not trigger intubation, as hypoxemia is often remarkably well tolerated in patients with COVID-19.^5^

Patients with COVID-19 differ in some ways from other patients with acute respiratory failure. On presentation to the ED, most have significant hypoxia without other organ failures and without hypercapnia. The interventions described here as well as the inclusion/exclusion criteria (Figure) are built upon recommendatons from the Intensive Care Society COVID-19 Guidance and Resource Library and designed specifically for patients with hypoxic respiratory failure.^6^

Supplemental oxygen is the mainstay of treatment of hypoxic patients, and for the majority of patients should begin with non-invasive maneuvers. If initial resuscitation strategies fail, if the patient becomes altered or shows continuing signs of respiratory failure, then he or she should be placed on mechanical ventilation. The remainder of this report describes our stepwise, non-invasive oxygen strategy for hypoxic patients with suspected or confirmed COVID-19.

### Step 3: Nasal Cannula

Initiate non-invasive oxygenation using a NC at 6 LPM. At room air, the air we breathe consists of 21% oxygen. A NC at 6 LPM equates to fraction of inspired oxygen (FIO_2_) of 44%.^7^ At rates greater than 6 LPM the laminar flow becomes extremely turbulent and oxygen being delivered at that rate is only as effective as 6 LPM. The dispersal of exhaled air at this rate has been measured to be 40 centimeters (cm), roughly 1.5 feet.^8^ This can be significantly higher in a patient in respiratory distress. We recommend placing a surgical mask over the NC as it has been shown to significantly reduce the dispersion distance.^9^

### Step 4: Nasal Cannula + Non-Rebreather

Should the patient continue to remain hypoxic or should their work of breathing increase as demonstrated by tachypnea, accessory muscle use, or change in mental status, the next step is to employ a NRB mask over the NC. The reservoir bag on the NRB should be at least two-thirds inflated before applying the mask to the patient. This will help to increase the amount of consistent FIO_2_ delivered. Oxygen via the NRB should be delivered at a rate of 15 LPM constituting a FIO_2_ of 70–80%.^10^

Air leak should be monitored and maintained, as these patients often have a minute ventilation far in excess of the 15 LPM from the NRB and the 6LPM of the NC combined; during respiratory distress, patients have a flow rate that varies widely between 30–120 LPM.^11^

### Step 5: Nasal Cannula + Non-Rebreather + Self-Proning

If the patient remains hypoxic or their work of breathing increases despite the NC and NRB, the next step in our non-invasive oxygenation strategy includes NC + NRB + self-proning. Proning patients has been shown to improve oxygenation, reduce respiratory effort, and decrease the need for intubation.^12^

Patients should rotate every 30–120 minutes from prone position to left-lateral decubitus, right-lateral decubitus, and upright sitting positions. A recent observational cohort study revealed that patients presenting to the ED during the COVID-19 pandemic with moderate to severe hypoxemia, periperal oxygen saturation (SpO_2_) 80% at triage demonstrated an improved SpO_2_ (94%) after five minutes of self-proning.^13^ In a case series of 15 awake patients with hypoxic respiratory failure, a series of short, two to four hour cycles of proning significantly improved oxygenation and was well tolerated by most patients.^14^

### Step 6: High-Flow Nasal Cannula + Non-Rebreather + Self-Proning

The next step in our non-invasive oxygenation strategy includes employing a HFNC in conjunction with a NRB and self-proning. HFNC warms oxygen to 37° Celsius creating 100% humidity, and when set to high flow rates 60 LPM can achieve FIO_2_ of nearly 100% and add about three to five cm H_2_O of positive-end expiratory pressure.^10^ A recent meta-analysis evaluating HFNC for COVID-19 patients with acute respiratory failure found a reduction in intubation, ICU admission, and mortality.^15^ Our strategy includes a NRB over the HFNC to assist in entrainment of O_2_ via oral inhalation. We also recommend self-proning of patients to improve O_2_ delivery and reduce respiratory effort.

## CONCLUSION

The novel coronavirus COVID-19 triggered a global pandemic leading to the infection of millions of individuals worldwide. Critically ill patients infected with COVID-19 often present to the ED with hypoxia and acute respiratory distress. The sheer scope and size of this pandemic has led to limited ventilator supplies and resources. Speaking with family members and determining wishes in patients who may not be good candidates for invasive ventilation is more time-consuming than ever before as most hospitals are limiting or banning visitors, and any procedure that can delay or prevent intubation has significant value.

With limited data and supplies during the COVID 19 pandemic, novel strategies have been stressed as a bridge therapy for the patients with hypoxemia and respiratory distress. Our case report describes a stepwise approach to providing non-invasive oxygenation for confirmed or suspected COVID-19 patients presenting to the ED with hypoxia and acute respiratory distress.

This strategy was developed during the initial days of the COVID-19 pandemic at a frontline hospital at the epicenter of the US outbreak. As in the case of our patient, this approach can be used to improve oxygen saturation, work of breathing, and reduce the need for early mechanical ventilation. Further evidence is needed to support causality and determine the effect this non-invasive has on disease severity and mortality.

## Figures and Tables

**Figure:**
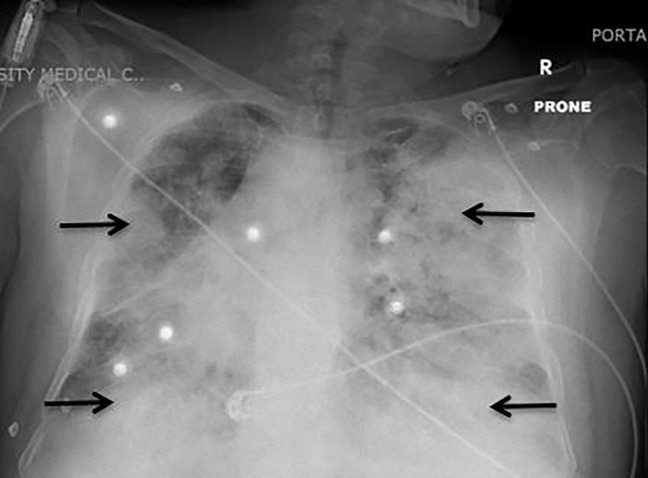
Non-Invasive Oxygenation Strategy for COVID-19 Patients with Acute Respiratory Distress. Inclusion and exclusion criteria based upon the recommendations from the Intensive Care Society (United Kingdom) and designed specifically for patients with hypoxic respiratory failure. *mmHg*, millimeters of mercury.

**Image:**
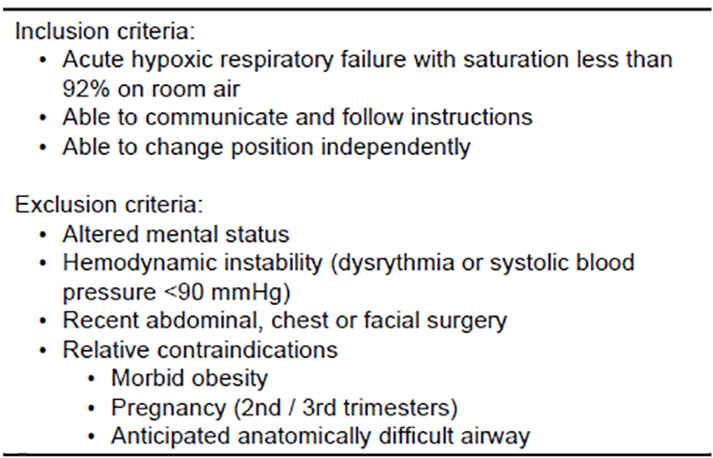
Chest radiograph (prone position) demonstrating bilateral patchy opacities, most prominently at periphery of the lung concerning for multifocal pneumonia (arrow).
